# Descriptions of acanthocephalans, *Cathayacanthus spinitruncatus* (Rhadinorhynchidae) male and *Pararhadinorhynchus magnus* n. sp. (Diplosentidae), from marine fish of Vietnam, with notes on *Heterosentis holospinus* (Arhythmacanthidae)

**DOI:** 10.1051/parasite/2018032

**Published:** 2018-07-23

**Authors:** Nguyen Van Ha, Omar M. Amin, Ha Duy Ngo, Richard A. Heckmann

**Affiliations:** 1 Department of Parasitology, Institute of Ecology and Biological Resources (IEBR), Vietnam Academy of Science and Technology 18 Hoang Quoc Viet Cau Giay Hanoi Vietnam; 2 Institute of Parasitic Diseases 11445 E. Via Linda 2-419 Scottsdale Arizona 85259 USA; 3 Department of Biology, Brigham Young University 1114 MLBM Provo UT 84602 USA

**Keywords:** Acanthocephala, *Cathayacanthus spinitruncatus*, *Pararhadinorhynchus magnus* n. sp., *Heterosentis holospinus*, Marine fish, Vietnam

## Abstract

Males of *Cathayacanthus spinitruncatus* Amin, Heckmann & Ha, 2014 (Rhadinorhynchinae Lühe, 1912) are described for the first time from *Leiognathus equulus* in Hai Phong and Nha Trang and from pony fish *Nuchequula flavaxilla* in Quang Ninh in the Pacific waters of Vietnam. The male allotype status is designated. Males of *C. spinitruncatus* are smaller and have fewer and smaller proboscis hooks and trunk spines than females. The male reproductive structures are in the posterior fifth of the trunk and with 6 club-shaped cement glands gradually merging into 6 independent cement gland ducts. The proboscis receptacle is more than half as long as the trunk and with a cephalic ganglion at its anterior end. In females, the receptacle is only about one fifth the length of the trunk. Specimens described as *Cathayacanthus bagarii* Moravec & Sey, 1989 were shown to have been wrongly assigned to *Cathayacanthus*. *Pararhadinorhynchus magnus* n. sp. (Diplosentidae) is described from *Scatophagus argus* off Hai Phong in the Gulf of Tonkin. It is the third species of the genus and is readily distinguished from the Australian species by having a considerably larger trunk and male reproductive structures, and more proboscis hooks. X-ray microanalysis (EDAX) of intact and gallium-cut hooks of *P*. *magnus* showed high calcium and phosphate mainly in the central core. Specimens of *Heterosentis holospinus* Amin, Heckmann & Ha, 2011 (Arhythmacanthidae) are also reported from *L. equulus* off Quang Binh, new host and locality records.

## Abstract (fr)

Les mâles de *Cathayacanthus spinitruncatus* Amin, Heckmann & Ha, 2014 (Rhadinorhynchinae Lühe, 1912) sont décrits pour la première fois chez *Leiognathus equulus* à Hai Phong et Nha Trang et chez *Nuchequula flavaxilla* à Quang Ninh dans les eaux du Pacifique au Vietnam. Le statut d’allotype mâle est désigné. Les mâles de *C. spinitruncatus* sont plus petits et ont des crochets du proboscis et des épines du tronc moins nombreux et plus petits que les femelles. Les structures reproductrices mâles sont dans le cinquième postérieur du tronc et ont 6 glandes cémentaires en forme de massue se fondant graduellement dans 6 conduits de glandes cémentaires indépendants. Le réceptacle du proboscis est plus long que la moitié du tronc et porte un ganglion céphalique à son extrémité antérieure. Chez les femelles, le réceptacle mesure seulement environ un cinquième de la longueur du tronc. Des spécimens décrits sous le nom de *Cathayacanthus bagarii* Moravec & Sey, 1989, ont été attribués à tort à *Cathayacanthus*. *Pararhadinorhynchus magnus* n. sp. (Diplosentidae) est décrit de *Scatophagus argus* au large de Hai Phong dans le golfe du Tonkin. Ceci est la troisième espèce du genre et on la distingue facilement des espèces australiennes par un tronc et des structures reproductives mâles considérablement plus grands, et de crochets plus nombreux sur le proboscis. La microanalyse aux rayons X (EDAX) des crochets intacts et coupés au gallium de *P. magnus* a révélé une teneur élevée en calcium et en phosphate, principalement dans le moyeu central. Des spécimens d’*Heterosentis holospinus* Amin, Heckmann & Ha, 2011 (Arhythmacanthidae) sont également signalés chez *L. equulus* au large de Quang Binh, ce qui constitue un nouvel hôte et une nouvelle localité.

## Introduction

Most of the recent taxonomic work on the Acanthocephala from Vietnam has been reported by the Amin-Heckmann-Ha team since 2000; see Amin & Ha [2, 3] and Amin et al. [4–7, 9–13]. Only three other species of *Rhadinorhynchus* and one species of *Gorgorhynchus* were previously reported from marine fishes in Vietnam by Arthur and Te [14].

Twenty species of acanthocephalans in 5 families were collected more recently from fishes in the Pacific and amphibians in central Vietnam, in 2016 and 2017. In the present report, we describe males of *Cathayacanthus spinitruncatus* Amin, Heckmann & Ha, 2014 (Rhadinorhynchidae) previously known from females only [13], a new species of the genus *Pararhadinorhynchus* Johnston & Edmonds, 1947 (Diplosentidae), and record *Heterosentis holospinus* Amin, Heckmann & Ha, 2011 in a new host and locality. For a better understanding of the chemical elements of hooks and their relationship to hook structure, intact and gallium-cut hooks of *P. magnus* were scanned with X-ray (EDAX) and analyzed for percent chemical elements in various parts of the hooks. This procedure has become a standard in our laboratory studies of the Acanthocephala [8, 19, 20].

## Materials and methods

### Collections

Eleven males and females of *C. spinitruncatus* were collected from two species of fish: the common pony fish *Leiognathus equulus* (Forsskål) in the northern Hai Phong area (20°51′54″N,106°41′2″E) and the southern Nha Trang coast (12°15′N,109°11′E) and from the yellow-spotted pony fish *Nuchequula flavaxilla* Kimura, Kimura & Ikejima off northern Quang Ninh Province (21°15′N,107°20′E). Two of 10 *L. equulus* from Hai Phong in 2016 and 1 from Nha Trang in October, 2017, and 7 of 20 *N. flavaxilla* from Quang Ninh were concurrently infected with *C. spinitruncatus* and other acanthocephalans. Specimens of *Pararhadinorhynchus magnus* n. sp. were collected from the spotted scat, *Scatophagus argus* (Linn.) (Scatophagidae) off Hai Phong in the Gulf of Tonkin in April 2015. Additionally, specimens of *Heterosentis holospinus* were collected from *L. equulus* off the same Quang Ninh Province as noted above in May 2017.

Freshly collected acanthocephalans were extended in water until proboscides were everted, and fixed in 70% ethanol for transport to our Arizona, USA laboratory for processing and further studies. Worms were punctured with a fine needle and subsequently stained in Mayer’s acid carmine, destained in 4% hydrochloric acid in 70% ethanol, dehydrated in ascending concentrations of ethanol reaching 100% (24 h each), and cleared in 100% xylene then in 50% Canada balsam and 50% xylene (24 h each). Whole worms were then mounted in Canada balsam. Measurements are in micrometers, unless otherwise noted; the range is followed by the mean values between parentheses. Width measurements represent maximum width. Trunk length does not include proboscis, neck, or bursa. Line drawing were created by using a Ken-A-Vision micro-projector (Ward’s Biological Supply Co., Rochester, New York, USA) which uses cool quartz iodine 150W illumination. Color-coded objectives, and 10×, 20×, and 43× lenses were used. Images of stained whole mounted specimens were projected vertically on 300 series Bristol draft paper (Starthmore, Westfield, Massachusetts, USA), then traced and inked with India ink. Projected images were identical to the actual specimens being projected.

### Scanning electron microscopy (SEM)

Four to six specimens that had been fixed and stored in 70% ethanol were processed for SEM following standard methods [23]. These included critical point drying (CPD) in sample baskets and mounting on SEM sample mounts (stubs) using conductive double-sided carbon tape. Samples were coated with gold and palladium for 3 min using a Polaron #3500 sputter coater (Quorum (Q150 TES) www.quorumtech.com) establishing an approximate thickness of 20 nm. Samples were placed and observed in an FEI Helios Dual Beam Nanolab 600 (FEI, Hillsboro, Oregon, USA) Scanning Electron Microscope with digital images obtained in the Nanolab software system (FEI, Hillsboro, Oregon, USA) and then stored on a USB for future reference. Samples were received under low vacuum conditions using 10 KV, spot size 2, 0.7 Torr using a GSE detector.

### Energy dispersive analysis for X-ray (EDAX)

Standard methods were used for preparation similar to the SEM procedure. Specimens were examined and positioned with the above SEM instrument which was equipped with a Phoenix energy-dispersive X-ray analyzer (FEI, Hillsboro, Oregon, USA). X-ray spot analysis and live scan analysis were performed at 16 kV with a spot size of 5 and results were recorded on charts and stored with digital imaging software attached to a computer. The TEAM *(Texture and Elemental Analytical Microscopy) software system (FEI, Hillsboro, Oregon, USA) was used. Data were stored on a USB. The data included weight percent and atom percent of the detected elements following correction factors, and were stored on a USB. All figures on the USB can be viewed by contacting the second author. The hooks were cut and scanned at two positions (Tip and Middle) with a gallium beam (LIMS) using a dual beam scanning electron microscope. The alignment of the hook prior to cutting generated a cross-section of the area.

### Ion sectioning of hooks

A dual-beam SEM with a gallium (Ga) ion source (GIS) is used for the LIMS (Liquid Ion Metal Source) part of the process. The gallium beam (LIMS) is a gas injection magnetron sputtering technique whereby the rate of cutting can be regulated. The hooks of the acanthocephalans were centered on the SEM stage and cross-sectioned using a probe current between 0.2 nA and 2.1 nA according to the rate at which the area is cut. The time of cutting is based on the nature and sensitivity of the tissue. Following the initial cut, the sample also goes through a milling process to obtain a smooth surface. The cut was then analyzed with X-ray at the tip, middle, and base of hooks for chemical ions with an electron beam (Tungsten) to obtain an X-ray spectrum. Results were stored with the attached imaging software then transferred to a USB for future use. The intensity of the GIS was variable according to the nature of the material being cut.

Type specimens were deposited in the University of Nebraska’s State Museum’s Harold W. Manter Laboratory (HWML) collection in Lincoln, Nebraska, USA.

## Results

Four of the 11 specimens of *C. spinitruncatus* collected were males. Female specimens in the same collection were identical to those described by Amin et al. [13] thus confirming the diagnosis of males. The 4 male and 7 female specimens were processed, studied and measured. Four males and five females of *P. magnus* n. sp. were collected from *S. argus* in April 2015. One male and five gravid females of *H. holospinus* were also found in 2 specimens of *L. equulus*.

### *Cathayacanthus spinitruncatus* Amin, Heckmann & Ha, 2014

urn:lsid:zoobank.org:act:C81AFB27-5691-4660-97B3-0001FB67AF4C

Family: Rhadinorhynchidae Travassos, 1923

Genus: *Cathayacanthus* Golvan, 1969

Type host: The common pony fish, *Leiognathus equulus* (Forsskål) (Leiognathidae).

Other host: The yellow-spotted pony fish *Nuchequula flavaxilla* Kimura, Kimura, Ikejima (Leiognathidae).

Type locality: The Hue City area south of Tonkin Gulf, Thua Thien, Hue Province, Central Vietnam (16°43′N,107°45′E), Vietnam.

Other localities: The northern Hai Phong area (20°51′54″N,106°41′2″E) and the southern Nha Trang coast (12°15′N,109°11′E) and off northern Quang Ninh Province (21°15′N,107°20′).

Type specimen: HWML collection no. 139405 (allotype male), no. 139406 (paratype male).

#### Description of males (Figs. 1–4)

**Figures 1–4.**
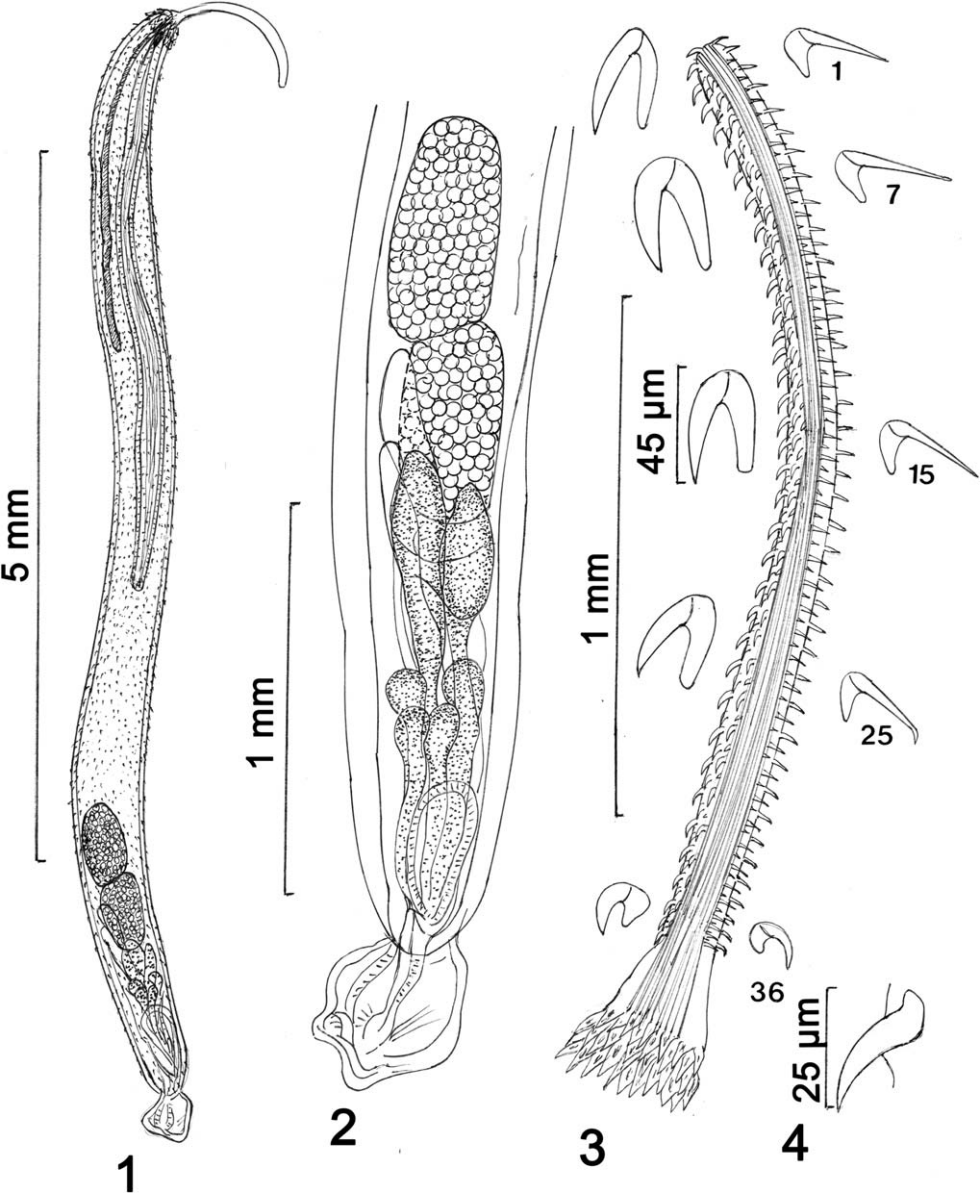
Line drawings of males of *Cathayacanthus spinitruncatus* from *Leiognathus equulus* in Vietnam. (1) A paratype male; note the very long proboscis receptacle compared to the trunk. The dots are trunk spines. (2) The reproductive system of specimens in Figure 1; note the posterior position of the reproductive system and the two larger anterior cement glands and the cluster of four posterior glands. Trunk spines not shown. (3) The proboscis of a male specimen and a representative sample of enlarged dorsal (right) and ventral (left) hooks numbered from anterior. (4) A trunk spine from the mid-trunk area.

Male (based on 4 mature adults): Rhadinorhynchidae. Specimens long, uniformly cylindrical, slightly arched, Trunk 3.92–7.75 (5.95) mm long by 0.36–0.60 (0.48) mm wide. Trunk totally spined (Fig. 1); spines with no dorsal-ventral differentiation. Anterior crown of spines (Fig. 3) in about 4–5 alternating rings of about 14 spines each posteriorly decreasing size and measuring 55 long anteriorly and 35 long posteriorly. Trunk spines (Fig. 4) in anterior half of trunk 20–25 long becoming slightly longer reaching 28 at posterior end. Proboscis long, perfectly cylindrical, curved dorsally, widest at posterior end, 1.14–2.38 (1.67) mm long by 0.06–0.11 (0.09) mm wide posteriorly with 14 longitudinal rows with 36–38 (37) hooks each and 1 lateral pair of sensory pits between posterior hooks. Hooks dorsoventrally asymmetrical (Fig. 3) with ventral and lateroventral hooks more robust and strongly recurved than straight, slender dorsal and laterodorsal hooks. Distal tip of posterior dorsal hooks curves slightly posteriorly (see hook no. 25, Fig. 3). Shape of dorsal and ventral hooks transition being intermediate laterally. Apical and subapical hooks relatively small becoming largest in anterior half of proboscis and gradually smaller and more crowded posteriorly. Basal hooks not specialized but slightly larger than smallest pre-basal hooks. Length and width (at base) of dorsal and ventral hooks nos. 1, 6, 12, 16, 20, 24, 28, 32, 36, and basal hooks in 1 male: Dorsal hooks: 27 (4), 30 (5), 35 (7), 32 (7), 30 (6), 27 (5), 27 (5), 22 (3), 17 (3), 18 (3). Ventral hooks: 37 (11), 42 (11), 45 (12), 45 (12), 42 (10), 40 (10), 27 (10), 22 (7), 15 (6), 20 (9). Hook roots simple, directed posteriorly, about as long as blades of ventral hooks but considerably shorter than blades of dorsal hooks. Neck 156 long by 114 wide posteriorly. Proboscis receptacle double-walled, gradually tapering posteriorly, much longer than proboscis and more than half as long as trunk, with cephalic ganglion at its anterior end, 2.60–3.92 (3.42) mm long by 0.17–0.18 (0.17) mm wide. Lemnisci multinucleated, subequal, markedly shorter than receptacle, 1.66–2.34 (2.01) mm long by 0.06–0.10 (0.08) wide. Reproductive system in posterior fifth of trunk. Testes elliptical, contiguous (Figs. 1, 2). Anterior testis 468–624 (550) long by 156–280 (232) wide, slightly larger than posterior testis 364–603 (489) long by 136–291 (239) wide. Sperm ducts prominent; common sperm duct 572–676 (624) long by 104–135 (119) wide. Six club-shaped cement glands in 1 large anterior pair and 1 cluster of 4 smaller posterior glands. Anterior glands overlap posterior testis, 360–437 (398) long by 220–229 (225) wide anteriorly. Posterior glands 260–270 (265) long by 94–198 (146) wide anteriorly. All cement glands gradually merging into independent cement gland ducts posteriorly connecting with posterior end of Saefftigen’s pouch 551–624 (587) long by 173–177 (175) wide anteriorly (Fig. 2). Bursa 260–447 (416) long by 208–364 (277) wide.

#### Remarks

**Figure 5.**
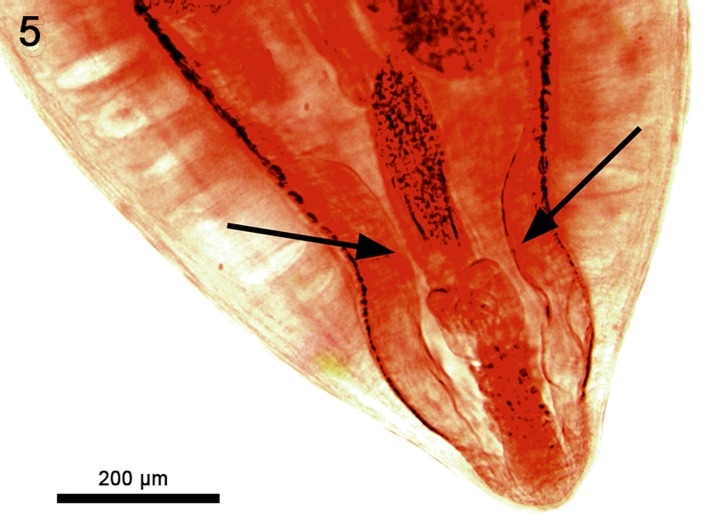
The posterior trunk of a female *Cathayacanthus spinitruncatus* from *Leiognathus equulus* in Vietnam showing two paravaginal muscular lobes (arrows).

The description of the males of *C. spinitruncatus* provides the only image of males of any valid member of the genus *Cathayacanthus*. The descriptions of *C. exilis* (Van Cleave, 1928) Golvan, 1969 and of *C. spinitruncatus* were based on 4 and 6 adult females from China and Vietnam, respectively. Males of *C. spinitruncatus* are considerably smaller (3.92–7.75 mm long) than females (14.27–33.07 mm long), with a smaller proboscis (1.82–2.39 compared to 2.25–2.60 mm) carrying fewer hooks per longitudinal row (36–38 compared to 53–61) that are shorter reaching 45 long ventrally and 35 dorsally, compared to 52 dorsally and ventrally in females. The pattern of dorso-ventral differentiation of hooks along the length of the proboscis is the same in both sexes. The proportion of lemnisci length to receptacle length was also comparable. However, in females, the receptacle was considerably shorter occupying about one fifth (23%) of trunk space (Fig. 23 of Amin et al. [13]) while it was over one half (57%) in males (Fig. 1). The description of the female reproductive system in Amin et al. [13], however, did not include two large paravaginal muscular lobes close to the body wall (Fig. 5, arrows).

### *Pararhadinorhynchus magnus* n. sp.

urn:lsid:zoobank.org:act:A7A57BB7-DBE1-4008-82A6-7CC53AA5CAE0

Family: Rhadinorhynchidae Travassos, 1923

Genus: *Pararhadinorhynchus* Johnston & Edmonds, 1947

Type host: *Scatophagus argus* (Linn.) (Scatophagidae)

Type locality: Off Hai Phong in the Gulf of Tonkin (20°51′54″N,106°41′2″E), northern Vietnam.

Type specimens: HWML collection no. 139410 (holotype male and paratypes on 1 slide), no. 139411 (allotype female), 139412 (paratypes).

Etymology: The new species is named for its larger body and other structures, especially the male reproductive system compared to those of the 2 other known species of the genus.

#### Description (Figs. 6–21)

**Figures 6–10.**
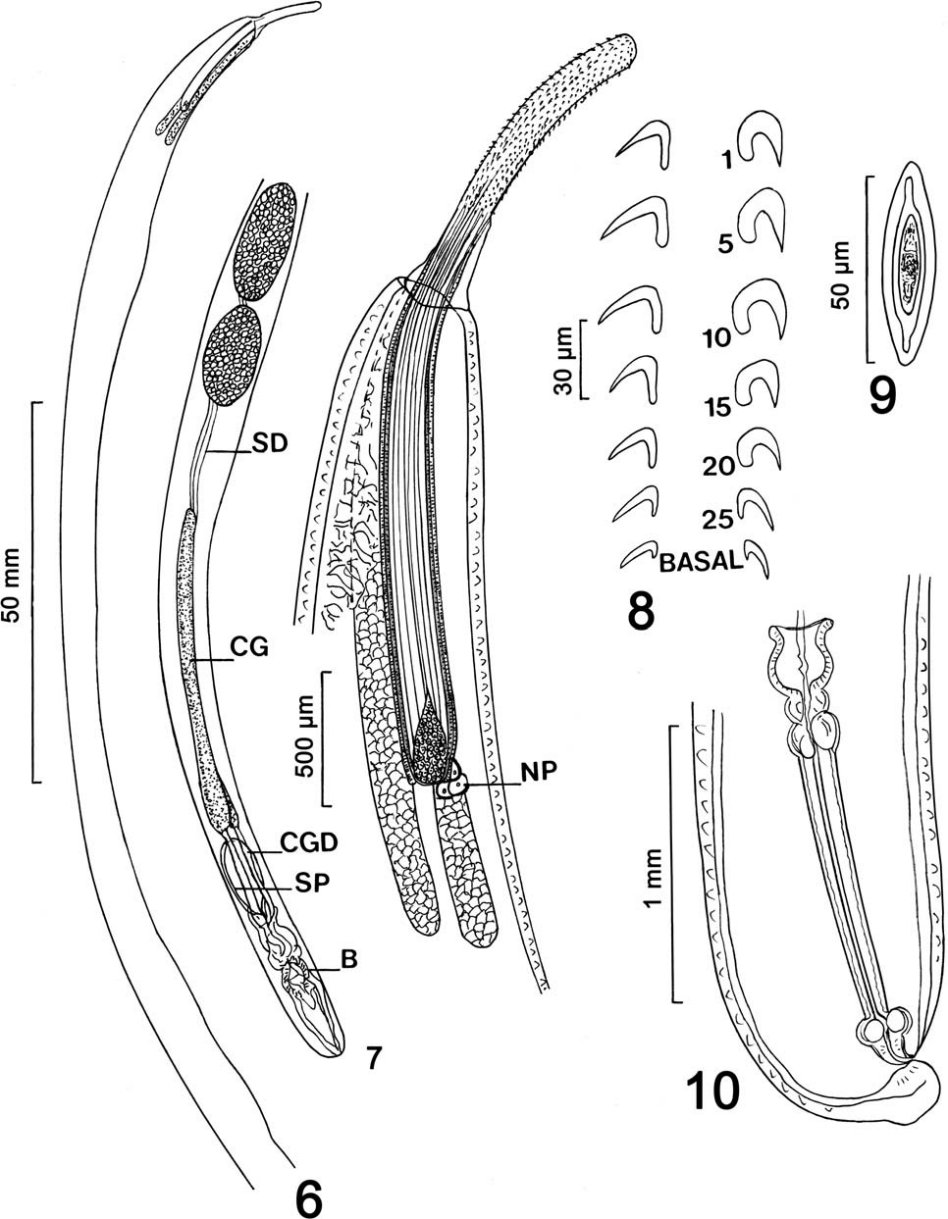
Line drawings of specimens of *Pararhadinorhynchus magnus* n. sp from *Scatophagus argus* in Vietnam. (6) Holotype male. Note that the testes are almost contiguous with each other but not with the tubular cement glands that are somewhat enlarged posteriorly. B: bursa; CG: Cement gland, CGD: cement gland duct; SD: sperm duct; SP: Saefftigen’s pouch. (7) The anterior trunk of the same male in Figure 6. Note the size relationships between the proboscis, receptacle and lemnisci, the reticular anastomoses of lacunar vessels, shape and size of cephalic ganglion, and the presence of a nuclear pouch (NP) at the posterior end of the receptacle. The lemnisci are shown of average length but can be about twice as long or occasionally about as long but thicker. (8) Selected dorsal (left) and ventral (right) hooks numbered from anterior. (9) Mature egg. (10) Female reproductive system. Note the plump dome-shaped terminal tip posterior to the subterminal gonopore.

**Figures 11–14.**
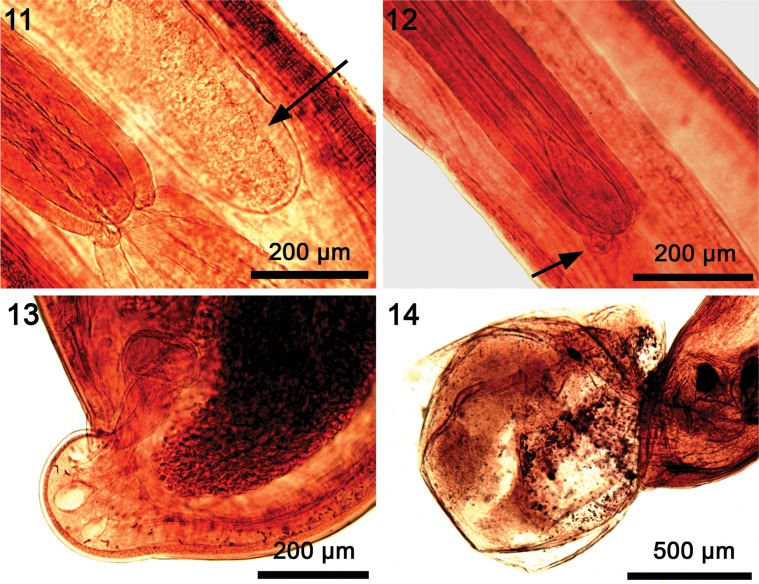
Microscopic images of specimens of *Pararhadinorhynchus magnus* n. sp. from *Scatophagus argus* in Vietnam. (11) The posterior portion of the proboscis receptacle showing the incomplete posterior outer wall of the receptacle and the clear external layer of a short lemniscus (arrow) slightly longer than the receptacle. (12) The posterior portion of another proboscis receptacle showing the nucleated cell pouch at its posterior end (arrow), position and shape of the cephalic ganglion, and part of a long lemniscus. (13) The posterior end of a gravid female showing the subterminal position of the gonopore anterior to the rounded posterior tip of the trunk, and the shape of the vagina. (14) The posterior end of a male specimen showing the rounded bursa lacking significant rays or sensory structures.

**Figures 15–21.**
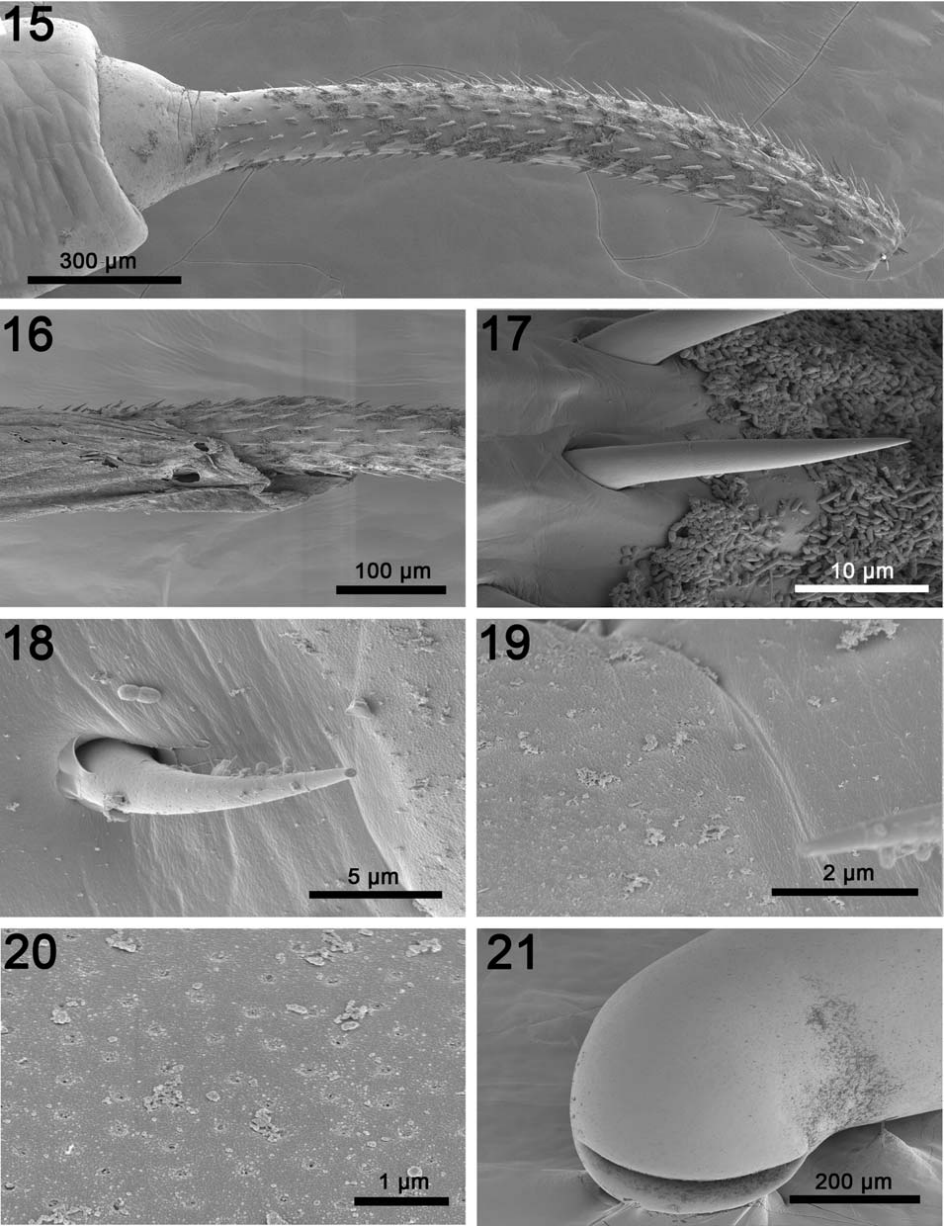
SEM of specimens of *Pararhadinorhynchus magnus* n. sp. from *Scatophagus argus* in Vietnam. (15) An evaginated proboscis showing the gradual decline in hook length posteriorly. Note the sharp posterior curvature of the ventral hooks and the less posteriorly curved dorsal hooks. (16) A proboscis deeply embedded in host intestinal wall showing a remaining part of the mucosal layer. (17) Proboscis hooks near the midsection of the proboscis. (18) A more sharply curved basal proboscis hook. (19) The transition between the proboscis showing no micropores (right) and the neck (left) with clear micropores. (20) Prominent widely spaced micropores on the mid-trunk; compare with neck micropores in Figure 19. (21) The posterior end of a male specimen showing the deep cleft marking the invaginated bursa.

*General*: With characters of the genus *Pararhadinorhynchus* (Rhadinorhynchidae). Trunk unarmed, long, uniformly cylindrical (Fig. 6) with prominent micropores of variable pore size and distribution in various parts of trunk and neck (Figs. 19, 20). Body wall with reticular lacunar system anastomoses with 2 main lacunar channels and many scattered nucleated cells (Fig. 7). Shared structures larger in females than in males. Proboscis long and cylindrical, widest posteriorly (Figs. 7, 15, 16), with many uniform hooks in 14–16 rows each with 23–27 hooks. Hooks largest subapically gradually decreasing in length posteriorly reaching smallest size basally (Figs. 17, 18). Hooks dorso-ventrally differentiated being more robust and more sharply curved with thicker roots ventrally, but slightly longer with more slender blades and roots, and less sharply curved dorsally. All hooks rooted. Hook roots simple, directed posteriorly, about as long as hooks anteriorly and at middle, becoming progressively more slender and shorter than blades posteriorly until reaching near vestigial state basally, but always evident (Fig. 8). Neck prominent, conical, widest posteriorly (Fig. 15). Proboscis receptacle double walled but outer wall incomplete posteriorly, widest anteriorly, about twice as long as proboscis, with large drop-shaped cephalic ganglion at its base and a nucleated pouch at its posterior ventral end (Figs. 7, 11, 12). Lemnisci equal, digitiform, plump, with definite clear cortical layer (Fig. 11), and of variable length; usually markedly longer than receptacle but occasionally as long as receptacle when considerably heavier (Figs. 7, 12).

*Male* (based on 4 mature adults with sperm; 1 male monorchid): See Table 1 for measurements and counts. Testes ellipsoidal, postequatorial, usually contiguous. Two long, tubular cement glands enlarged posteriorly and non-contiguous with posterior testis anteriorly (Fig. 6). Two sperm ducts visible in space between anterior cement gland and posterior testis measuring 0.31–1.5 (0.81) mm long by 0.04–0.08 (0.07) mm wide. No such space in trunk of monorchid male crowded with large testis measuring 3.12 long by 0.75 wide. Cement gland ducts prominent, relatively wider posteriorly, surrounding Sarfftigen’s pouch and common sperm duct (Fig. 6). Bursa plump, rounded, with very few rays and no evident special features or sensory structures, but with occasional typical body wall nucleated cells (Fig. 14). Posterior end of trunk occasionally with deep cleft marking completely invaginated bursa (Fig. 21).

**Table 1.** Morphometric comparisons among species of *Pararhadinorhynchus.*

	*P. mugilis*		*P. coorongenis*		*P. magnus* n. sp.	
Characters	Johnston & Edmunds [22]		Edmunds [15]		This paper	
	Males	Females	Males	Females	Male	Females
Trunk size (mm)	3.1–11.4 × 0.23–0.61	3.9–19.2 × 0.22–0.69	7.0–11.0 × 0.40–0.70	9.0–15.0 × 0.45–0.55	22.50–43.75 (35.22) × 0.60–1.20 (0.93)	57.00–71.25 (63.17) × 0.77–1.37 (1.14)•
Proboscis size	900 × 200	900 × 200	500–580 × 0.12–0.23	510–520 × 0.15–0.22	648–950 (840) × 95–130 (108)	915–1170 (1025) × 100–125 (112)
Hook rows/per row	18/16–17	18/16–17	14–16 (16)/8–10 (9)	14–16 (16)/8–10 (9)	15–16 (15.25)/22–25 (24.50)	14–16 (15.25)/24–27 (25.50)
Longest & smallest hooks	21 & 5 (Fig. 11)	21 & 5 (Fig. 11)	50 & 25 (Fig. 2)	50 & 25 (Fig. 2)	——–	35 & 22
Hook roots	Shorter than blades, post. hooks rootless	Shorter than blades, post. hooks rootless	As long as blades, post. 4 hook rootless	As long as blades, post. 4 hook rootless	As long as blades, reduced posteriorly	As long as blades, reduced posteriorly
Neck size	0.15–0.25 × ——–	0.15–0.25 × ——–	150 × ——–	150 × ——–	166–208 (192) × 166–260 (246)	187–208 (198) × 229–239 (232)
Receptacle size (mm)	0.61–1.3 × 0.12–0.20	0.61–1.3 × 0.12–0.20	0.6–0.8 × 0.18–0.26	0.6–0.8 × 0.18–0.26	1.30–2.18 (1.86) × 0.16–0.25 (0.21)	1.40–2.25 (1.91) × 0.19–0.24 (0.21)
Cephalic ganglion size	——–	——–	——–	——–	187–260 (213) × 20–78 (58)	230–364 (299) × 73–83 (78)
Lemnisci size (mm)	0.80 × ——– (as long as recept.)	0.80 × ——– (as long as recept.)	Twice as long as receptacle	Twice as long as receptacle	Twice or same as long as receptacle	Twice or same as long as receptacle
Ant. testis size (mm)	0.28–1.1 × 0.08–0.24	N/A	0.45–1.05 × 0.35–0.45	N/A	1.12–2.20 (1.82) × 0.62–1.07 (0.91)	N/A
Post. Testis size (mm)	0.27–1.1 × 0.08–0.23	N/A	0.50–0.9 × 0.34–0.43N/A	1.25–2.25 (1.89) × 0.57–0.87 (0.75)	N/A	
Cement gland size (mm)	0.45–2.5 × ——–	N/A	2, long & slender	N/A	4.25–10.62 (7.52) × 0.17–0.25 (0.22) and 0.25–0.37 (0.32)	N/A
Cement gl. Duct size (mm)	——–	N/A	——–	N/A	1.07–1.75 (1.36) × 0.11–0.18 (0.14)	N/A
Safftigen’s pouch size (mm)	——–	N/A	——–	N/A	1.04–1.62 (1.27) × 0.26–0.37 (0.30)	N/A
Bursa size (mm)	——–	N/A	——–	N/A	0.75–0.87 (0.81) × 1.00	N/A
Fem. reproductive syst.	N/A	——–	N/A	2.8–3.4	N/A	1.60–1.72 (1.66)
Vagina length	N/A	150 (ganglionic complex ?)	N/A	——–	N/A	0.19–0.21 (0.20)
Uterus length (mm)	——–	1.1–1.4	N/A	——–	N/A	0.88–1.09 (0.99)
Uterine bell length	N/A	200	N/A	——–	N/A	260–340 (300)
Uterus glands area length	N/A	——–	N/A	——–	N/A	0.21–0.27 (0.24)
Egg size	N/A	52–62 × 13–18	N/A	42–46 × 8–10	N/A	52–65 × 15–18
Gonopore	Terminal	Terminal	Terminal	Terminal	Terminal	Subterminal
Host	*Mugil cephalus*	*Mugil cephalus*	*Aldrichetta forsteri*	*Aldrichetta forsteri*	*Scatophagus argus*	*Scatophagus argus*
Geography	Australia	Australia	Australia	Australia	Vietnam	Vietnam

• Range (mean) measurements are in micrometers unless otherwise specified.

*Female* (based on 5 mostly gravid specimens): See Table 1 for measurements and counts. One female demonstrating dorso-ventral differentiation of proboscis hooks comparing length followed by thickness at base of selected numbered dorsal (D), and ventral (V) hooks from anterior: hook # 1: 25, 6 (D), 25, 8 (V); # 5: 35, 10 (D), 32, 13 (V), # 10: 32, 7 (D); 30, 10 (V); # 15: 31, 7 (D), 27, 10 (V); # 20: 27, 7 (D), 25, 9 (V); # 25: 25, 5 (D), 25, 7 (V); basal: 22, 5 (D), 22, 6 (V). Eggs ellipsoidal elongate with polar prolongation of fertilization membrane (Fig. 9). Gonopore subterminal anterior to prominent rounded posterior tip (Figs. 10, 13).

#### X-ray scans (EDAX)

**Figure 22.**
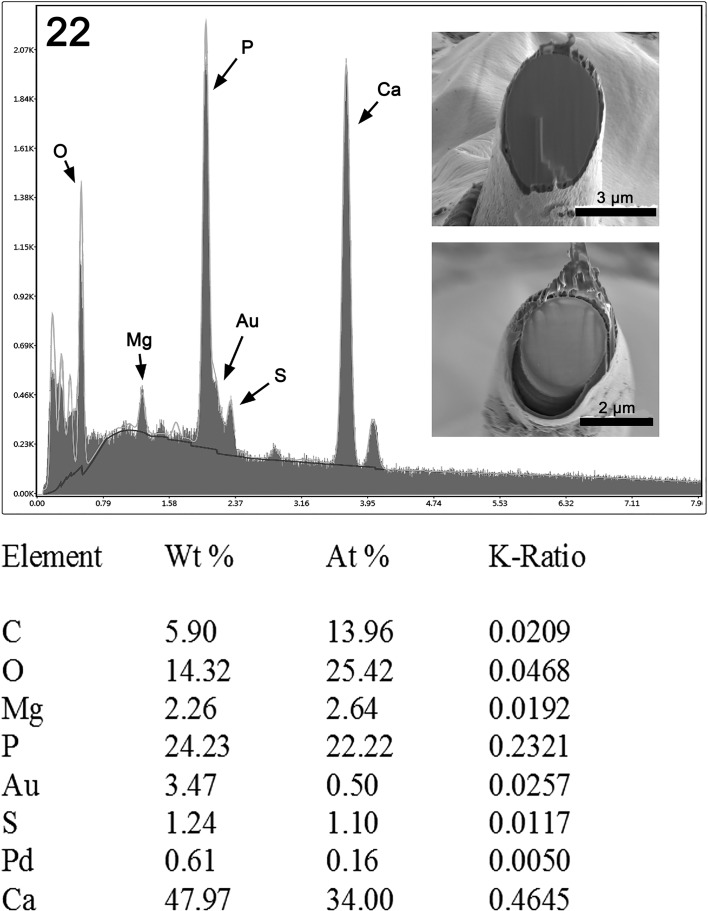
X-ray elemental scan (XEDS) of a specimen of *Pararhadinorhynchus magnus* n. sp. hook. Center area of a gallium cut showing high phosphorus and calcium content; see Table 2. Insert: SEM of cross gallium cut hook.

The results of the X-ray scans using EDAX are listed in Table 2 and Figure 22. A definite layering was visible with high sulphur content in the outside layer.

#### Remarks

Johnston & Edmonds [22] characterized their new genus *Pararhadinorhynchus* as being *Rhadinorhynchus*-like by having 2 long tubular cement glands, a long proboscis with numerus hooks, and a double walled proboscis receptacle. In *Pararhadinorhynchus*, however, the cephalic ganglion is normally at the base of the receptacle, the trunk is aspinose, and basal proboscis hooks are not enlarged or projecting at a right angle to the proboscis. These features are well represented in *P. mugilis* described from the flathead gray *Mugil cephalus* Linn. (Mugilidae) in South Australia [22] and *Pararhadinorhynchus coorongensis* Edmonds 1973 also collected from South Australia from the yellow-eyed mullet *Aldrichetta forsteri* (Cuvier & Valenciennes) (Mugilidae) as well as those of *P. magnus* n. sp. from Vietnam. Additionally, all three species usually have contiguous testes and cement glands distant from posterior testis especially in *P. mugilis*, similar eggs, and similar proboscis hook blades and shape of trunk, lemnisci and reproductive structures. The lemnisci in *P. mugilis* were about as long as the receptacle but about twice as long in *P. coorongensis*. In *P. magnus*, they were variable but often markedly longer than the receptacle; they were shorter when more plump.

*Pararhadinorhynchus coorongensis* primarily differs from *P. mugilis* and *P. magnus* by having a considerably smaller proboscis with markedly fewer hooks and smaller eggs (Table 1). The marked dorso-ventral differentiation of proboscis hooks noted in our specimens was not reported in either one of the two Australian species. However, we suspect that this was simply overlooked. Line drawings of hooks of *P. mugilis* (Fig. 11 of Johnston & Edmonds, 1947 [22]) appear to represent dorsal hooks, while those of *P. coorongensis* (Fig. 2 of Edmonds, 1973 [15]) represent ventral hooks, especially the anterior and middle hooks. Posterior hooks in the same figures lack roots, which is also probably an oversight because of their minute size. In our specimens from the spotted scat *S. argus* from a different family (Scatophagidae) in Vietnam, the male reproductive system is markedly larger than in the two Australian species. In addition, the female gonopore in *P. magnus* is decidedly subterminal behind a prominent round posterior tip but terminal in the two Australian species.

### About *Heterosentis holospinus*

The 1 male and 5 gravid females were practically identical to those described by Amin et al. [12]. The rooted apical and larger subapical proboscis hooks, the 3–4 posterior unrooted spine-like hooks, the unspined anterior trunk cone, the receptacle shape and plump longer lemnisci, and trunk shape and size were almost identical. The male reproductive structures, especially the shape of the thick sperm ducts, the rounded bursa, and the shape and position of the testes and cement glands were very similar. The female reproductive system especially the complex vagina, the prominent and widening uterus, the uterine bell with many cells, and the eggs were also very similar. However, the nucleated pouch at the posterior end of the receptacle was not consistently prominent and the trunk spines were faint and less readily visible.

#### Specimens

HWML coll. No. 139404 (voucher specimens on one slide).

## Discussion

Golvan [18] relegated *Rhadinorhynchus exilis* Van Cleave, 1928 from the crucian carp, *Carassius carassius* (Lin.) in China to his new genus *Cathayacanthus* based on the absence of large specialized basal proboscis hooks as is typical in *Rhadinorhynchus*. The proboscis of Golvan’s [18] new genus characteristically exhibited dorso-ventral differentiation of hooks, the cephalic ganglion was positioned at the anterior end of the receptacle, and the trunk was spinose. Trunk spines were only anterior in *C. exilis* but covered the whole trunk in *C. spinitruncatus*. Both species were described from females only. The present description of males provides for the first time a description of males of any member of the genus with its characteristic reproductive system.

This latter feature becomes especially significant in the diagnosis of other acanthocephalans incorrectly assigned to the genus *Cathayacanthus* such as *Cathayacanthus bagarii* Moravec & Sey, 1989 which was collected from *Bagarius bagarius* (Hamilton) (Sisoridae), a freshwater fish from the Red River near Hanoi. Specimens of *C. bagarii* were distorted with the anterior end of all described specimens withdrawn and the proboscis totally inverted inside the receptacle. Moravec & Sey [25] dismissed the importance of “alleged dorsoventral asymmetry of proboscis hooks.” They also indicated the position of the cephalic ganglion to be in the “posterior half” of the receptacle. Amin et al. [13] provisionally retained that species in *Cathayacanthus* until more information becomes available. That information has now become available. The reproductive system in our specimens demonstrates the presence of 6 club-shaped cement glands (Figs. 1, 2). The male specimens of *C. bagarii* had “Four very long tubular cement glands” (Fig. 6A of Moravec & Sey, [25]) as well as posterior cephalic ganglion and no asymmetry in proboscis hooks which does not agree with the pattern in *Cathayacanthus* as we know it. Golvan [18] doubted that males of *Cathayacanthus* would have 4 cement glands as presumably found only in Rhadinorhynchidae. We therefore remove the provisional status of *C. bagarii* and declare that it belongs in another genus. Before the present material of *C. spinitruncatus* became available, and based on Moravec & Sey [25], we [13] erroneously indicated in the generic diagnosis that males of *Cathayacanthus* have 4 tubular cement glands.

*Pararhadinorhynchus magnus* n. sp. is the third species of the genus described from a new family of marine fish, Scatophagidae, in northern Vietnam. The other two species, *P. mugilis* and *P. coorongensis*, were described from two species of mugiliform fishes in South Australia. Findings demonstrate new host and geographical distributional records of species of *Pararhadinorhynchus*, but within the Pacific. *Mugil cephalus* and *Aldrichetta forsteri* are distributed in coastal tropical and subtropical waters worldwide [16, 24]. *Scatophagus argus* is distributed around the Indo-Pacific region, to Japan, New Guinea, and southeastern Australia [17]. The above host distribution would not preclude the finding of *P. magnus* or other species of the genus in new geographical locations within that range of geographical distribution. The distance between northern Vietnam and southern Australia reported in this study is certainly suggestive.

Previous articles have included the use of X-ray scans (EDAX) in the analysis of acanthocephalan hooks or attachment structures [8, 20, 21]. The gallium cut hooks of *P. magnus* showed high phosphorus and calcium content in the mid-cut center of the hook (Table 2). X-ray analysis in conjunction with a scanning electron microscope (Fig. 22) has been important for evaluating both living and non-living materials for the presence of chemical elements [1, 19]. The result is an intact surface that can be scanned with X-ray. These results were compared with other studies published by our laboratory [8] and are considered diagnostic fingerprints of taxonomic relevance.

**Table 2.** X-ray scans (XEDS) for hooks of *Pararhadinorhynchus magnus*. including Gallium cuts (wt.%)*.

Elements	Hook	Tip cut	Mid cut center	Mid cut edge
Magnesium (Mg)	0.53	0.88	2.26	1.65
Phosphorus (P)	6.57	11.67	24.23	14.67
Sulfur (S)	3.08	1.68	1.24	3.53
Calcium (Ca)	14.15	25.17	47.97	27.33

* Four chemical elements are listed in weight percent (wt.%) for area. Common elements in living cells (H.O.N.) and coating and cutting elements (Pd, Au, Ga) present but not listed.

Specimens of *H. holospinus* are recorded from a new host, *L. equulus* (family Leiognathidae) which was concurrently infected with other acanthocephalans, in a new locality off Quang Ninh at the Gulf of Tonkin near Halong bay, where the type specimens were originally collected. *Heterosentis holospinus* is apparently more widespread in fish from at least two percid families in the Halong Bay area.

### Conflict of interest

The authors declare that they have no conflict of interest.
